# Giant piezoresponse in nanoporous (Ba,Ca)(Ti,Zr)O_3_ thin film†

**DOI:** 10.1039/d3sc06712b

**Published:** 2024-05-20

**Authors:** Motasim Billah, Yukana Terasawa, Mostafa Kamal Masud, Toru Asahi, Mohamed Barakat Zakaria Hegazy, Takahiro Nagata, Toyohiro Chikyow, Fumihiko Uesugi, Md. Shahriar A. Hossain, Yusuke Yamauchi

**Affiliations:** a Australian Institute for Bioengineering and Nanotechnology (AIBN), The University of Queensland Brisbane QLD 4072 Australia md.hossain@uq.edu.au y.yamauchi@uq.edu.au; b School of Mechanical and Mining Engineering, Faculty of Engineering, Architecture and Information Technology (EAIT), The University of Queensland Brisbane QLD 4072 Australia; c Kagami Memorial Research Institute for Materials Science and Technology, Waseda University 2-8-26 Nishiwaseda, Shinjuku-ku Tokyo 162-0051 Japan; d Faculty of Advanced Science and Technology, Kumamoto University 2-39-1 Chuo-ku, Kurokami, Kumamoto-shi Kumamoto 860-8555 Japan terasawa@cs.kumamoto-u.ac.jp; e Department of Life Science & Medical Bioscience, School of Advanced Science and Engineering, Waseda University 2-2 Wakamatsu-cho, Shinjuku Tokyo 162-8480 Japan; f Department of Chemistry, Faculty of Science, Tanta University Tanta 31527 Egypt; g Research Center for Electronic and Optical Materials, National Institute for Materials Science (NIMS) 1-1 Namiki Tsukuba Ibaraki 305-0044 Japan; h Center for Basic Research on Materials, National Institute for Materials Science (NIMS) 1-1 Namiki Tsukuba Ibaraki 305-0044 Japan; i Research Network and Facility Services Divisioin, National UInstitute for Materials Science (NIMS) 1-2-1 Sengen Tsukuba Ibaraki 305-0047 Japan; j Department of Plant and Environmental New Resources, Kyung Hee University 1732 Deogyeong-daero, Giheung-gu Yongi-si Gyeonggi-do 446-01 South Korea; k Department of Materials Process Engineering, Graduate School of Engineering, Nagoya University Nagoya 464-8603 Japan

## Abstract

Lattice strain effects on the piezoelectric properties of crystalline ferroelectrics have been extensively studied for decades; however, the strain dependence of the piezoelectric properties at nano-level has yet to be investigated. Herein, a new overview of the super-strain of nanoporous polycrystalline ferroelectrics is reported for the first time using a nanoengineered barium calcium zirconium titanate composition (Ba_0.85_Ca_0.15_)(Ti_0.9_Zr_0.1_)O_3_ (BCZT). Atomic-level investigations show that the controlled pore wall thickness contributes to highly strained lattice structures that also retain the crystal size at the optimal value (<30 nm), which is the primary contributor to high piezoelectricity. The strain field derived from geometric phase analysis at the atomic level and aberration-corrected high-resolution scanning transmission electron microscopy (STEM) yields of over 30% clearly show theoretical agreement with high piezoelectric properties. The uniqueness of this work is the simplicity of the synthesis; moreover the piezoresponse *d*_33_ becomes giant, at around 7500 pm V^−1^. This response is an order of magnitude greater than that of lead zirconate titanate (PZT), which is known to be the most successful ferroelectric over the past 50 years. This concept utilizing nanoporous BCZT will be highly useful for a promising high-density electrolyte-free dielectric capacitor and generator for energy harvesting in the future.

## Abstract 2

Nanoporous Ba_0.85_Ca_0.15_(Ti_0.9_Zr_0.1_)O_3_ (BCZT) films, as lead-free piezoelectrics, exhibited a *d*_33_ ∼10 times higher than nonporous BCZT, making them promising for high-density dielectric capacitors and energy harvesters.

## Graphical abstract

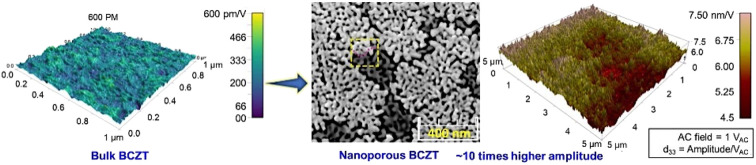

## Introduction

Piezoelectric materials have been widely used in sensors, transducers, undersea sonar, and small devices for medical diagnostic applications. Researchers have not previously deeply examined the possibility of energy harvesting using piezoelectric devices as a rival to large-scale traditional energy sources, such as coal, hydrocarbons, and other known sources.^1^ The key component in the design of piezoelectric generators is the use of highly efficient, stable, environmentally friendly, and low-cost piezoelectric materials that can respond effectively to applied mechanical stress that distorts the crystal structure and electric voltage generation. The most rigorous work has been done on lead zirconate and lead titanate, Pb(Zr,Ti)O_3_ (PZT), with various doping of a variety of composites, with a high piezoelectric charge constant (*d*_33_) (650 pC N^−1^) and electromechanical coupling coefficient (*k*_33_) (0.78), in a direction longitudinal to the applied stress.^2^ For half a century, the PZT ceramic family has been representative of a large class of technologically important materials—that is, piezoelectrics—that convert mechanical stress (strain) and electrical voltage (charge), representing the piezoelectric effect and the inverse piezoelectric effect.^3^ The highest *d*_33_ (∼2500 pC N^−1^) and *k*_33_ (>0.9)^4^ have been reported in prototypes of Pb-based relaxors, Pb(Mg_1/3_Nb_2/3_)O_3_-PbTiO_3_ (PMN-PT) and Pb(Zn_1/3_Nb_2/3_)O_3_-PbTiO_3_ (PZN-PT), which are only in the form of a single crystal.^5^ These highly toxic lead-based materials are commercially dominant piezoelectric materials. However, researchers worldwide have been searching for lead-free piezoelectric materials for more than a decade, aiming to replace these toxic materials.^6^ This goal—that is, the invention of a Pb-free piezoelectric material with a performance equivalent to or even superior to that of PZT-based piezoelectric materials—has not yet been fulfilled. In recent years, barium titanate (BaTiO_3_, BTO),^7^ bismuth sodium titanate (Bi_0.5_Na_0.5_TiO_3_, NBT),^8^ potassium sodium niobate (K_0.5_Na_0.5_NbO),^9^ and bismuth ferrite (BiFeO_3_)^10^ have been widely studied as Pb-free sustainable materials. In particular, BTO has shown potential for use in piezoelectric applications, such as multilayer ceramic capacitors, as it has a high dielectric constant and low dielectric loss.^11^ However, the values of *d*_33_ (191 pC N^−1^) and *k*_33_ (0.494) in BTO^12^ are still far behind those of its commercially available Pb-based counterparts.

Consequently, researchers worldwide have attempted to improve the performance of BTO. Cao *et al.* reported that a reduction in the grain size to 1.6 μm is responsible for enhancing *d*_33_ to a value as high as 460 pC N^−1^.^13^ More recently, Wada *et al.* synthesized grain-oriented ceramics of BTO by the templated grain growth (TGG) method, obtaining a remarkable *d*_33_ value of 788 pC N^−1^.^14^ However, the TGG method requires complex remixing and recalcination of spherical and flake-structured presynthesized BTO in different ratios. This gives the desired planar orientation (111)—where the dipole rotation happens to be in its least energy-invasive state to transfer surface charge, resulting in the best piezoelectric response. In another report, a seed-passivated texturing process was utilized to fabricate textured PZT ceramics through microplatelet templating.^15^ Though this strategy achieved a piezoelectric coefficient *d*_33_ of 760 pC N^−1^, it involves multi-step preparation. By considering all these complex and extended synthesis methods, a simple synthesis approach with a very high yield is required to prepare a new BTO candidate that is highly competitive with commercially available Pb-based materials.

Many efforts to dope BTO with different elements have demonstrated its potential to compete with commercially available PZT. A calcium and zirconium co-doped barium titanate system (Ba(Ti_0.8_Zr_0.2_)O_3_-(Ba_0.7_Ca_0.3_)TiO_3_, BZT-BCT) with a noticeably enhanced *d*_33_ (∼620 pC N^−1^) was also reported by Liu *et al.*^16^ The orthorhombic Ba_0.85_Ca_0.15_(Ti_0.9_ Zr_0.1_)O_3_ (BCZT) in its morphotropic phase boundary (MPB) is thermodynamically unstable in a ferroelectric phase, with polarization rotation occurring within the minimum energy state in the crystal system, and it can be easily manipulated by external stress or an electric field with minimum effort, yielding the best ferroelectric properties. In addition, according to some earlier publications on BCZT polycrystalline ceramics, the coexistence of polymorphic phase transitions can play a significant role in enhancing the dielectric and piezoelectric properties.^16–18^ The variations in the composition of Ca (14–18 mol%) trigger the polymorphic phase (the coexistence of orthorhombic-tetragonal phases), tending toward ferroelectric relaxor behavior. Consequently, a wide range of piezoelectric and ferroelectric properties can be tailored in such novel single BCZT structural models by simply varying their stoichiometry. The highest dielectric constant *ε*_r_ (5000) has been reported in bulk BCZT by varying the Ca content between 12 and 16 mol%.^19^ As another strategy, our previous study on nanoporous films hinted that the existence of elongated nanopores certainly leads to a successful strain engineering process for enhancing piezoelectricity. Meanwhile, the improved surface area enhances the dipole moment at the surface, thereby enhancing the piezoelectric response.^20^

Hence, we prepared highly strained nanoporous Ba_0.85_Ca_0.15_(Ti_0.9_Zr_0.1_)O_3_ (BCZT) by a soft-templating method and we investigated the strain pattern developed in the BCZT film. We also studied the atomic-scale strain mapping using high-resolution electron microscopy and analyzed it by using the presence of strong Bragg reflections in its Fourier transform. In principle, strain and local deformation can be determined directly by measuring the displacement of the lattice fringes on the atomic scale. The technique of quantitative measurement of such displacement and strain field analysis in this study is based upon a theoretical method developed by M. J. Hytch,^2,9^ regarded as geometric phase analysis. Piezoresponse force microscopy (PFM) study is also popular for confirming piezoelectricity in the nano-regime and to calculate the piezoelectric coefficient (*d*_33_).^11,21^ The PFM study in this work gathers an accurate vertical piezoresponse (*d*_33_) mapping, a hysteresis-butterfly loop for quantitative analysis. It is found that the obtained *d*_33_ and strain values in the synthesized nanoporous BCZT are in good agreement and indicate ten-fold higher *d*_33_ compared to its non-porous bulk counterpart. Such a nanoporous film shows great promise for high-density energy harvesting.

## Results and discussion

### Synthesis of nanoporous BCZT film and observation of porous structure

**Fig. 1:**
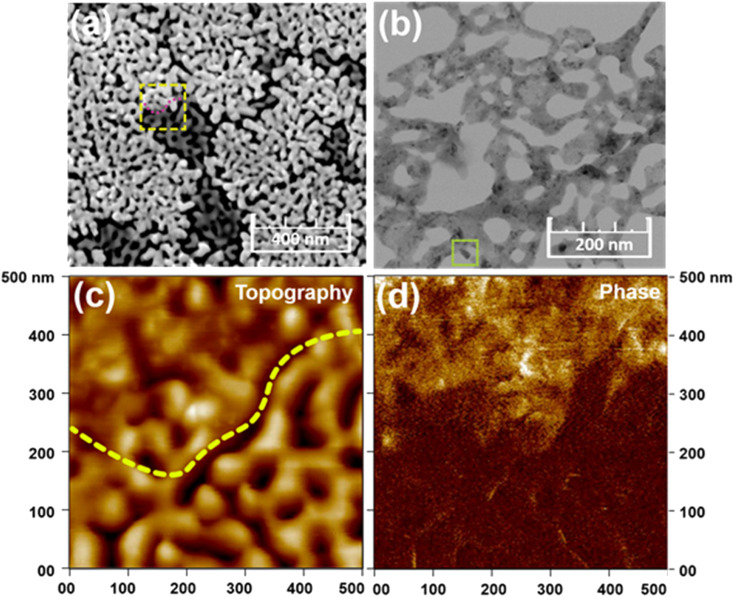
Electron micrograph images of nanoporous BCZT. (a) Scanning electron microscope (SEM) image showing surface morphology. The boundary between highly crystallized and amorphous/poorly crystallized phases is indicated by a square. (b) Scanning transmission electron microscope (STEM) image showing pore walls. (c and d) Atomic force microscopy (AFM) mapping: (c) topographical image showing highly crystallized and amorphous/poorly crystallized phases, and (d) phase image for distinguishing between these two phases.

Nanoporous BCZT films were synthesized by a sol–gel-based method using a diblock copolymer (PS-*b*-PEO) as a pore-directing agent. The role of PS-*b*-PEO as a pore-directing agent was demonstrated in our previous report.^22^ In the precursor solution, the PS-*b*-PEO polymers form spherical micelles (*i.e.*, a template) where the PS blocks are the core and the PEO blocks are the shell. The resulting nanoporous BCZT presents both highly crystallized and amorphous/poorly crystallized phases, as evidenced by the different morphologies (light and dark) in the SEM image (Fig. 1a). The growth architecture of the pores on the top of the film surface calcined at 650 °C is elongated due to the framework crystallization, which is further verified by the STEM image (Fig. 1b). The coexistence of two phases is also further confirmed by the difference in stiffness in the phase images from atomic force microscopy (AFM) (Fig. 1c and d).^19^ The boundary between the two phases is clearly separated by the dotted line marked in the topographical image in Fig. 1c. The difference in contrast is evident in the region with high contrast as a highly crystalline region, whereas low contrast indicates the amorphous/poorly crystalline region. Without the use of a pore-directing agent, the bulk BCZT (calcined at 700 °C) possesses random grains of several tens of nanometers in size (Fig. S1a†).

On the other hand, with the use of PS-*b*-PEO, nanopores start to form after calcination at 400 °C due to the removal of the template (Fig. S2a†). The nanopores become large by joining with their neighbouring pores and are elongated as the calcination temperature increases (Fig. S2b†). Crystallinity can hardly be observed in the films calcined at 400 °C and 500 °C (Fig. S2c†). Abundant robust nanopores with highly crystallized frameworks can obviously be observed at 650 °C (Fig. S1b, c and S2c†). The expansion and partial collapse of the nanopores occurs in a temperature range higher than 650 °C. Therefore, 650 °C is fixed as the optimized temperature. From the above results, it is found that the resulting nanoporous architecture shows a very different morphology from the BCZT bulk film (it should be noted that we attempted to synthesize a bulk film without PS-*b*-PEO at a calcination temperature of 650 °C, but no significant XRD peaks were found, indicating amorphous BCZT; therefore, the calcination temperature was increased to 700 °C (Fig. S1c†).

As shown in Fig. S1c,† both bulk and nanoporous BCZT films show a perovskite phase. In the case of the BCZT bulk film, some peaks other than those of BCZT (*i.e.*, impurities) are observed in the low-angle region. However, the addition of PS-*b*-PEO can facilitate a homogeneous mixing of inorganic sources without precipitation, thereby forming an almost pure BCZT phase. The average crystallite sizes of the bulk and nanoporous BCZT roughly calculated from the Scherrer equation are *ca*. 10 nm and 23 nm, respectively. It is interesting that nanoporous BCZT becomes more crystallized with the addition of a soft template, and the calculated crystallite size is smaller than the thickness of the pore walls (Fig. 1b).

### Atomic distribution in nanoporous BCZT film

**Fig. 2:**
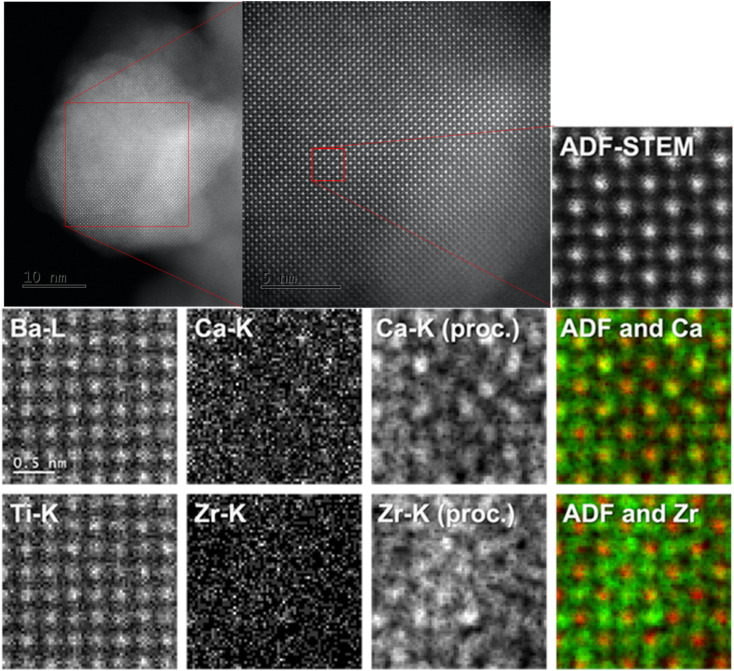
ADF-STEM image and EDS mappings for nanoporous BCZT. A high-resolution ADF-STEM image with low-index crystal band axes ([100] or [001]) magnified from the low-magnification ADF-STEM image. The raw EDS intensity mappings for Ba, Ti, Ca and Zr, which are indicated by Ba-L, Ti-K, Ca-K, and Zr-K, respectively. The processed EDS intensity mappings for Ca and Zr, which are indicated by Ca-K (proc.) and Zr-K (proc.), respectively. EDS maps (green) of Ca or Zr overlaid with the ADF-STEM image (red), which are indicated by ADF and Ca, and ADF and Zr, respectively.

The ADF-STEM image and EDS mappings for nanoporous BCZT are shown in Fig. 2. The low-magnification ADF-STEM image shows that this sample is a collection of crystal grains of less than 100 nm. A high-resolution ADF-STEM image of the grains shows low-index crystal band axes ([100] or [001]). It can be deduced that the strong-intensity columns are Ba and the weak ones are Ti, since this is an ADF-STEM image, in which the intensity is approximately proportional to the square of the elemental number. The EDS mappings are obtained from the same area as the ADF-STEM images. Since the Ba and Ti peaks appear at almost the same energy position in the EDS spectra (Fig. S3†), they are also almost identical in the map. Therefore, the columns of Ba and Ti cannot be distinguished from the EDS mapping. However, it can be said that the previous assumption of Ba in the intense column and Ti in the less intense column is correct by combining the ADF-STEM and EDS results. Additionally, the substitution positions for Ca and Zr can be estimated by comparing the high-resolution ADF-STEM images and the EDS mappings. It is difficult to identify the positions of Ca and Zr due to their weak intensity. Therefore, image processing is performed to enhance the visibility of each position. These images are superimposed on the high-resolution ADF-STEM images. The results show that Ca and Zr are substituted for Ba and Ti. These mapping images are direct evidence of the successful doping of Ca and Zr atoms. The Ba, Ca, Ti, and Zr compositional ratio coincides with the ICP result, supporting the formation of Ba_0.85_Ca_0.15_(Ti_0.9_Zr_0.1_)O_3_. The ADF-STEM images for nanoporous BTO are shown in Fig. S4.† The details of the doping levels are evaluated from XPS (Fig. S5†).

### Measurement in PFM analysis

Quantitative studies of piezoelectric behavior and polarization-related properties at the nanoscale can be challenging and extremely sensitive to the experimental conditions of piezoresponse force microscopy (PFM). However, near-accurate results can be achieved by carefully optimizing several factors. These include (i) electrostatic contributions due to cantilever-surface nonlocal and tip-surface local capacitive forces, (ii) tip bias to calibrate and compensate for linear dielectric contributions, and (iii) choice of driving voltage (*V*_AC_) and bias window of measurement.^23^ In the measurement process, the electrostatic contribution can be represented by the equation: *A*_el_ = *G*(*ω*)(*V*_DC_ − *V*_surf_), where *A*_el_ represents the electrostatic contribution, *G*(*ω*) denotes the frequency-dependent electromechanical response function, *V*_DC_ is the direct current voltage, and *V*_surf_ represents the surface potential. Unlike the piezoelectric contribution to the signal, which remains independent of the tip bias for linear piezoelectric or dielectric components, the electrostatic contribution varies linearly with bias and becomes zero when the null condition *V*_DC_ = *V*_surf_ is met. This null condition can be identified by carefully assessing the uniformity of the grayscale contribution in the amplitude image in PFM. In an unoptimized image, surface charge (non-piezoresponse components) may appear as very bright contrasts (white highlights). The proportionality factor *G*(*ω*) is strongly dependent on frequency and can significantly amplify the contribution to the PFM signal, particularly at frequencies near 300 kHz.^24^

**Fig. 3:**
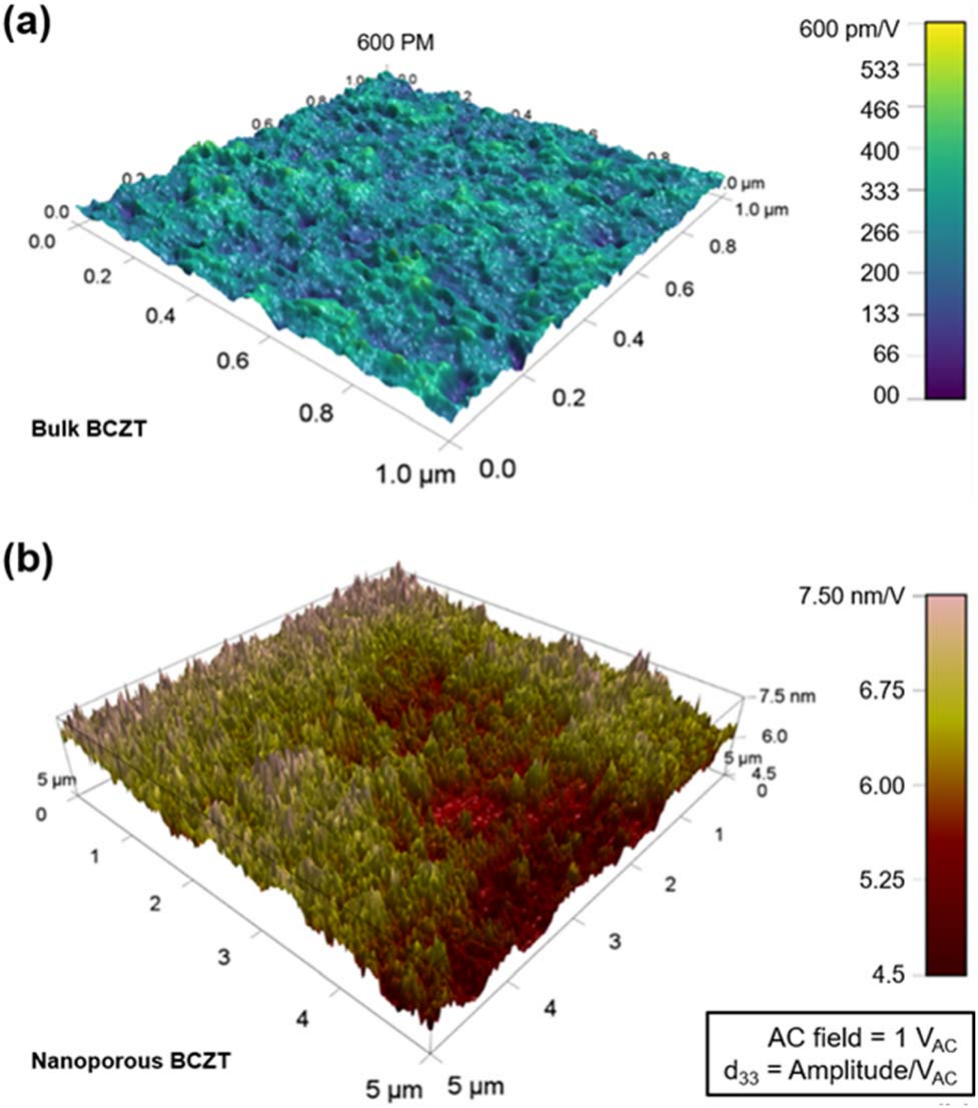
Amplitude distribution of *d*_33_ mapping for (a) bulk and (b) nanoporous BCZT. The mapping areas for the bulk and nanoporous samples are 1 × 1 μm^2^ and 5 × 5 μm^2^, respectively. Approximately 10 times the amplitude is observed for nanoporous BCZT compared to bulk BCZT.

A heavily doped (conductive)-silicon cantilever was used with a spring constant of 2.7 N m^−1^ and medium stiffness with high contact resonance, and fairly tight adhesion to the sample surface (essential for compensating for local capacitive forces) by the cantilever was found to be best suited for all studied materials including the reference sample. A contact resonance of 200–300 kHz was present in the studied sample during scanning. A test reference sample of PZT film (the same film thickness as the studied sample) with known *d*_33_ was taken for which the contact resonance lay within the same frequency range and the *V*_AC_ modulation window of the cantilever was tuned to resemble the known piezoresponse (*d*_33_) of PZT to compensate for the proportionality factor *G*(*ω*) due to any frequency artefact that may have occurred. 1 V was found to be stable and was kept constant in all studied samples since all the samples were fairly responsive below this threshold. Therefore, sample deformation becomes *A*_out-plane_ = *d*_33_ at 1 V (ref. 11) and the amplitude image of PFM becomes a direct representation of the vertical piezoresponse (*d*_33_), as shown by the plotted data in Fig. 3. Generally, the unit for *d*_33_ is C N^−1^ or m V^−1^.

### Piezoresponse and strain in nanoporous BCZT film

**Fig. 4:**
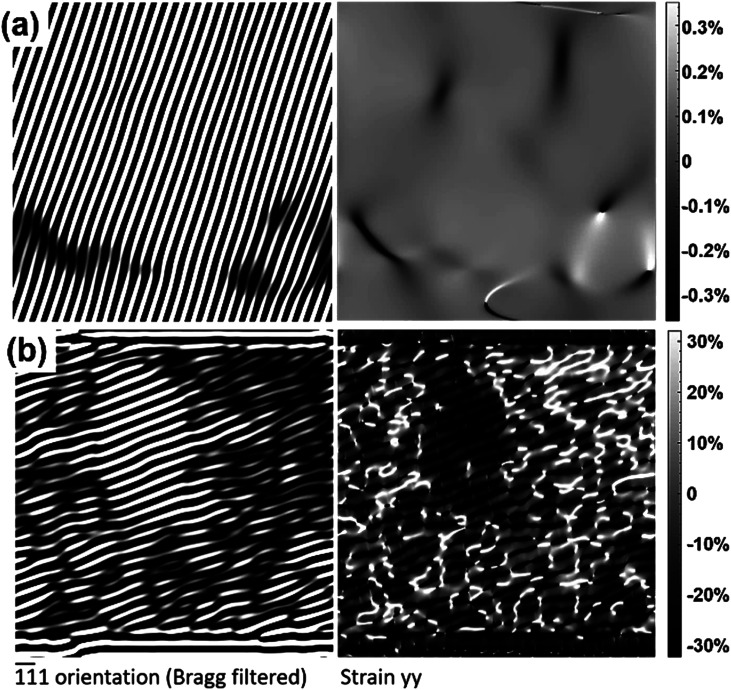
Bragg-filtered image (left) and strain field image (right) for (a) bulk and (b) nanoporous BCZT films. The bar on the right-hand side of the strain field images represents the degree of “strain”.

The mapping of piezoresponse, representing vertical amplitudes with respect to the film substrate, is illustrated in Fig. 3. The amplitudes for the bulk (Fig. 3a) and nanoporous films (Fig. 3b) go from 0 to 600 pm V^−1^ and from 4.5 to 7.5 nm V^−1^, respectively. Therefore, approximately 10 times the amplitude is observed for nanoporous BCZT compared to that of the bulk. The maximum amplitude for the bulk film is 600 pm and that for the nanoporous film is 7.5 nm. These results are reflected in the *d*_33_ values at 1 V in the amplitude–voltage loops, which will be discussed later. This is because the amplitude in Fig. 3 indirectly means *d*_33_, as a voltage of 1 *V*_AC_ is applied in the PFM experiment. Surprisingly, the result is that this large remnant *d*_33_ value of nanoporous BCZT far exceeds those of other Pb-based materials.^2,4,7–10,12–16^ The strain is calculated with high-resolution scanning transmission electron microscopy (HR-STEM) (Hitachi HF5000 aberration-corrected STEM). A Bragg-filtered image of the lattice strain is calculated using the FFT patterns (see details in the ESI;† the validity of strain values and Fig. S6†). Fig. 4 shows the strain field images for bulk and nanoporous BCZT films.

**Fig. 5:**
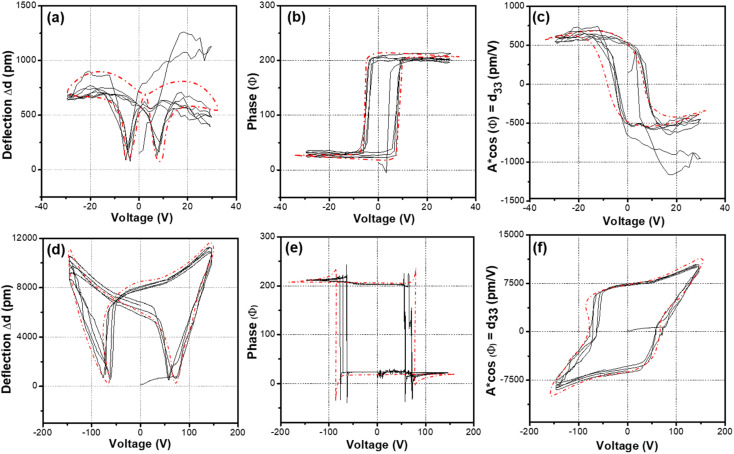
Remanent piezoresponse (off-field). (a) Deflection–voltage hysteresis loop, (b) phase–voltage hysteresis loop, (c) *d*_33_–voltage hysteresis loop of bulk (non-porous) BCZT; and (d) deflection–voltage hysteresis loop, (e) phase–voltage hysteresis loop, and (f) *d*_33_–voltage hysteresis loop for nanoporous BCZT. The averaged normalized plot (red dotted line) was compiled from four iterations (black lines), which correspond to four sets of data obtained during nano-indentation probing at the same location.

Surprisingly, there is a large difference in the calculated strain distribution between bulk and nanoporous BCZT films. The values are ≥0.3% and over ∼30% for bulk and nanoporous BCZT films, respectively. The strain for the nanoporous BCZT film is approximately 100 times larger than that of bulk BCZT film. The mathematical relationship between strain (obtained quantitatively from geometric phase analysis; Fig. 4) and piezoresponse (obtained from PFM; Fig. 5) shows they are directly related where our quantitative findings satisfy the bounded relation equation, as follows:*ε* = *d*_33_*EG*(*ω*)^−1^,where *ε* is strain, *d*_33_ is the vertical piezoresponse, *E* is the coercive field, *G*(*ω*)^−1^ is a proportionality factor calibrated for an optical beam deflection sensor (OBD) equipped with a DART PFM, provided by the PFM manufacturer Oxford Instruments, USA and given in the ESI† (Fig. S6).

### Piezoresponse in nanoporous BCZT film

The nano-indented piezoelectric properties were evaluated with the amplitude–voltage, phase–voltage, and piezoelectric hysteresis loops from piezoresponse force microscopy (PFM) (Oxford Instruments Cypher High Voltage Dual AC Resonance Tracking; DART) under off-field conditions. Fig. 5 shows the averaged normalized plot (red dotted line), compiled from four iterations, which correspond to four sets of data obtained during nano-indentation probing at the same location. This approach allows for an extended duration of “polling” under the applied electric field compared to a single probing or iteration. Also, it ensures the repeatability and quality of the collected data. The relationship between deflection and voltage for bulk (non-porous) and nanoporous BCZT is depicted by deflection–voltage hysteresis loops (Fig. 5a and d). The amplitude values for the nanoporous BCZT film are approximately ten times larger (7500 pm V^−1^*vs.* 550 pm V^−1^) than those for the bulk BCZT film (Fig. 5c and f). In the case of the nanoporous BCZT film, the coercive voltage *V*_c_ at the cross-point of the loop is approximately ±50 V, which is indicative of a larger internal strain (since the strain is directly proportional to *d*_33_) compared to the bulk BCZT film (Fig. 5f). Meanwhile, the relationship between phase and voltage for nanoporous BCZT is depicted by rectangular loops with a squareness ratio near unity, which are absent in non-porous BCZT (Fig. 5b and e) due to the high energy content possessed by nanoporous BCZT. 180° phase flips are observed for both nanoporous and bulk films (Fig. 5b and e). The *d*_33_–voltage hysteresis loops, which are ferroelectric polarization–electric field-like hysteresis loops, can be observed (Fig. 5c and f). The remnant *d*_33_ value is 7500 pm V^−1^ (Fig. 5f). This value of remnant *d*_33_ is more than 10 times larger than that for the bulk BCZT film and for the previously reported BZT-BCT.^15^ Compared to those of non-doped BTO and PZT, *i.e.*, representative Pb-based materials, the value of *d*_33_ for nanoporous BCZT is much larger.^2,12^ Furthermore, the obtained value in this study is demonstrated to be reasonable according to *ε*_r_ (Fig. S7†). The large dielectric constant (*ε*_r_ = 1500 and 1000 for nanoporous and BCZT, respectively) and adequately low leakage current density (*J* = 10^−6^ A cm^−2^ for nanoporous BCZT in comparison to 10^−7^ A cm^−2^ for bulk BCZT) for practical use are confirmed in both nanoporous and bulk films (Fig. S8†). Moreover, the dielectric loss tan *δ* values for both nanoporous and bulk films are almost the same at approximately 0.03 to 0.04 in the frequency range from 0.001 to 0.01 MHz, whereas they show an increase above 0.01 MHz (Fig. S7†). In addition, the tan *δ* value for the nanoporous BCZT film shows a more gradual increase than that for the bulk BCZT film. It is approximately 0.12 at 1 MHz, which is approximately half that for the bulk film. Therefore, higher *ε*_*r*_ and lower tan *δ* for the nanoporous film than for the bulk film are observed over this frequency range. In these measurements, the piezoresponse is not symmetrical along the vertical axes, indicating a strain memory effect. This effect is microscopically attributed to domain switching in non-180° domains which was previously reported in PZT.^25^ Additionally, the values of the coercive voltages of *V*_c−_ and *V*_c+_ are not the same in the *d*_33_–voltage hysteresis loops, which indicates the existence of an internal bias voltage. The calculated values of *V*_c−_ and *V*_c+_ in the *d*_33_–voltage hysteresis loops for bulk BCZT are ≅−3.5 V and ≅8.2 V, respectively (Fig. 5c), while for nanoporous BCZT they are ≅−62.8 V and ≅57.5 V, respectively (Fig. 5f). Such asymmetry is remarkable in the deflection–voltage hysteresis loop (Fig. 5a and d). Two distinct values are observed at 0 V, indicating a significant internal bias voltage. This is due to the offset voltage being applied by the doping and porous structure. The coercive field, *V*_c_ (Fig. 5f), is notably five times higher than that of its bulk counterpart (Fig. 5c). Additionally, the energy generation equation, 
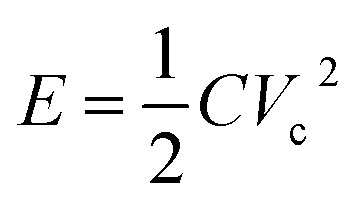
, highlights the significance of the “square” term of *V*_c_, serving as another breakthrough indicator for mainstream energy harvesters, alongside the discovery of the massive *d*_33_. Such a phenomenon is not observed in bulk BCZT materials.

## Conclusions

We have investigated the piezoelectric properties of a nanoporous BCZT film with artificially engineered strains at the nanoscale using a soft-templating approach. Deformation arises from the pore wall structure, leading to strain verified through geometric phase analysis. As is evident from the PFM measurements, the *d*_33_ of nanoporous BCZT is 10 times greater than that of bulk BCZT. The introduction of the porous structure causes strong anisotropic stress on the oxygen octahedron in the BCZT crystal, which significantly distorts the crystal lattice. As a result, spontaneous polarization occurs in a particular orientation, which is thought to have resulted in large piezoelectricity. Our nanoporous BCZT film demonstrates significant piezoelectricity solely through the introduction of a nanoporous structure, eliminating the necessity for creating intricate architectures, such as nanowires, nanofibers, or nanocore/shell structures.^26–28^ This study introduces a new approach for the cost-effective production of Pb-free piezoelectric materials using much lower annealing temperatures while achieving an ultra-high piezoresponse, *d*_33_. Furthermore, it opens up a new avenue for high-density energy harvesting applications, serving as a renewable alternative to fossil fuels—an aspect that has not previously been explored.

## Data availability

Data are available from the authors upon reasonable request.

## Author contributions

The concept for the experiment was initially developed by M. B., M. S. A. H. and Y. Y. Nanoporous BCZT was synthesized by M. B., M. K. M. and Y. T. XPS, dielectric constant, dielectric loss, leakage current density, *etc.* were conducted by M. B. under the supervision of M. S. A. H. and Y. Y. ADF-STEM was performed and analyzed by T. A., Y. T., M. B. Z. H., T. N., T. C. and F. U. M. B. Z. H., M. K. M. and Y. T. wrote the manuscript. All authors discussed the results at all stages and participated in the development of the manuscript.

## Conflicts of interest

The authors declare no conflict of interest.

## Supplementary Material

SC-015-D3SC06712B-s001
