# Supplementary material for: Giant piezoresponse in nanoporous (Ba,Ca)(Ti,Zr)O3 thin film
Source: Chem Sci. 2024 May 20;15(24):9147–54. doi: 10.1039/d3sc06712b (PMC11186322; doi:10.1039/d3sc06712b)
Supplement: SC-015-D3SC06712B-s001 [file SC-015-D3SC06712B-s001.pdf]

This journal is © The Royal Society of Chemistry 2024

Experimental section

Synthesis. BCZT films on a 0.5 wt.% Nb-doped strontium titanate ($SrTiO_3$, STO) substrate (Shinkosha, Japan) were prepared using a sol-gel-based spin-coating process. The film prepared without a pore-directing agent is denoted a "bulk BCZT film". For the preparation of the bulk BCZT film, barium acetate, $Ba(C_2H_3O_2)_2$ (0.85 g), and calcium acetate, $Ca(C_2H_3O_2)_2$ (0.1 g), were dissolved and stabilized in separate glacial acetic acid solutions. Both solutions were mixed and heated at 50 °C for 5 min to generate a transparent solution. An additional solution of a certain amount of titanium butoxide, $Ti(OC_4H_9)_4$ (0.9 g; dissolved in toluene), was added dropwise to the above solution, followed by the dropwise addition of zirconium butaoxide, $Zr(OC_4H_9)_4$ (0.1 g), with continuous stirring for 5 min to obtain a homogeneous solution. For the preparation of the nanoporous BCZT film, a diblock copolymer solution containing PS_{18000}-*b*-PEO_{7500} (50 mg) as a pore-directing agent dissolved in tetrahydrofuran (1.5 mL) was slowly added dropwise to the above solution. The PS-*b*-PEO block polymeric micelles can serve as a pore-directing agent to form uniformly sized nanopores. Bulk and nanoporous BTO films without Ca and Zr atoms were also prepared under the same conditions except for the use of Ca and Zr precursors. The resulting precursor solutions were spin-coated on the substrate for 30 sec. at a spinning rate of 3000 rpm to form a film layer with a thickness of approximately 200 nm. The ellipsometry characterization confirmed that a film thickness of 170 nm was obtained. The substrate was then annealed at 120 °C for 5 min, calcined at designated temperatures with a ramping rate of 1 °C min^{-1}, and held for 10 min at the highest temperature.

Materials characterizations. The surface structures of the composites were observed with field-emission scanning electron microscopy (FESEM) (Hitachi SU7001 SEM). Images were collected with a low-voltage Hitachi SU7001 SEM at an accelerating voltage of 5 kV and a current of 10 mA. The crystallinity of the composites was evaluated with wide-angle X-ray diffraction (XRD) (Rigaku RINT 2500X). The XRD pattern was collected with monochromated Cu Kα radiation (40 kV and 40 mA) at a scanning rate of 0.5 °C min^{-1}. Scanning transmission electron microscopy (STEM) for nanoporous BTO and BCZT films was performed using equipped with a Cs corrector in the irradiation and imaging system (JEOL JEM-ARM200F). TEM was equipped with two EDS detectors and a total solid angle was 0.98 Sr at an acceleration voltage of 200 kV. The drift correction function was used in the high-resolution EDS measurements. TEM samples were prepared by peeling the films from substrates using a cutter knife and dispersing them in ethanol. Following dispersion, the suspension was dropped onto a Cu mesh with a carbon support film. The doping levels in the nanoporous BCZT film were determined using X-ray photoelectron spectroscopy (XPS) (Kratos AXIS-ULTRA DLD).

PFM amplitude distributions. The amplitude distributions were observed using high-voltage piezoresponse force microscopy (HV-PFM) (Cypher DART, Asylum Research). A heavily doped conductive silicon cantilever with a spring constant of 2.7 N $\cdot m^{-1}$ was used. Its medium stiffness with high contact resonance and fairly tight adhesion to the sample surface (essential for compensating local capacitive forces) were found to be the best suited of all the studied materials. The sample deformation becomes $A_{out-plane} = d_{33}$ at $V_{AC} =$ 1 V [1], and the amplitude image of PFM becomes a direct representation of the vertical piezoresponse (d_{33}), as plotted in **Figure 5**.

Strain study. The strain for bulk BCZT was calculated with high-resolution scanning transmission electron microscopy (HR-STEM) (Hitachi HF5000 aberration-corrected STEM). We have taken the geometric phase approach for this purpose, which is based on combining real-space and Fourier-space information. By studying the amplitude of an electron micrograph image, the local contrast of the fringes and their phase represent their positions. Consequently, the strain can be measured by calculating the local Fourier components of the lattice fringes in an image, following Hÿtch *et al*. [2].
Measurement of dielectric properties. The dielectric constant (ε_r) was measured using a semiconductor parameter analyzer (Keysight, B1500A Semiconductor Device Parameter Analyzer). A 0.5 wt.% Nb-doped STO substrate was used as the bottom electrode, and platinum (Pt) was sputtered as the top electrode ($\emptyset \cong 100\ \mu m$) on the synthesized film. A DC voltage of 3 mV was applied to the synthesized nanoporous and bulk films with a thickness of 170 nm. ε_r was measured at 298 K under the above conditions, and the dielectric loss ($\tan\delta$) was calculated using Equation (S1):

$$\tan\delta = \frac{1}{2\pi C_p R_p} \qquad \text{(S1)}$$

Validity of the strain values. Here, the strain values of BCZT were calculated to ensure the validity of the measured piezoelectric properties in this study by comparing the strain values estimated from TEM analysis in this study. The strain values were calculated using Equation (S1), where ε, d_{33}, E and $G(\omega)$ are the electric field-induced strain, piezoelectric constant, electric field and proportionality factor of strain for the applied voltage, respectively [3].

$$\varepsilon = \frac{d_{33}E}{G(\omega)} \qquad \text{(S2)}$$

The value of $G(\omega)$ was determined from the calibration plot (**Figure S6**) of the effective piezoelectric sensitivity obtained from an optical beam deflection sensor equipped with PFM and was strongly

frequency-dependent and can amplify the contribution to the PFM signal at a frequency near 300 kHz [3]. It was estimated that an amplification factor from 10 to 100 $pm\,V^{-1}$ might arise subject to the optimization of the electrostatic contributions, local capacitive forces, and tip bias, as broadly explored for the contact resonance between the sample and tip from 200 to 300 kHz. Therefore, the value of $G(\omega)$ was set to 10. The values of electric field-induced strain were calculated to be $\cong$0.003 ($\cong$0.3%) and $\cong$0.245 ($\cong$25%) for the bulk and nanoporous films, respectively, using $G(\omega) = 10$ and the calculated values from the piezoelectric hysteresis loops in **Figure 4**. They are in good agreement with the values of $\varepsilon \geq$ 0.3% and over ~30% for the bulk and nanoporous films estimated from TEM analysis, respectively. Therefore, it is considered that the evaluation of piezoelectric properties in this study was reasonable.

Comment on leakage current. The leakage current was measured using a semiconductor parameter analyzer (Keysight, B1500A Semiconductor Device Parameter Analyzer). A Si/5 wt.% Nb-doped STO substrate was used, and titanium (Ti)/Pt was sputtered as the bottom electrode. A voltage of 1 V was applied to the synthesized films with a thickness of 170 nm. The measurement temperature was 298 K.

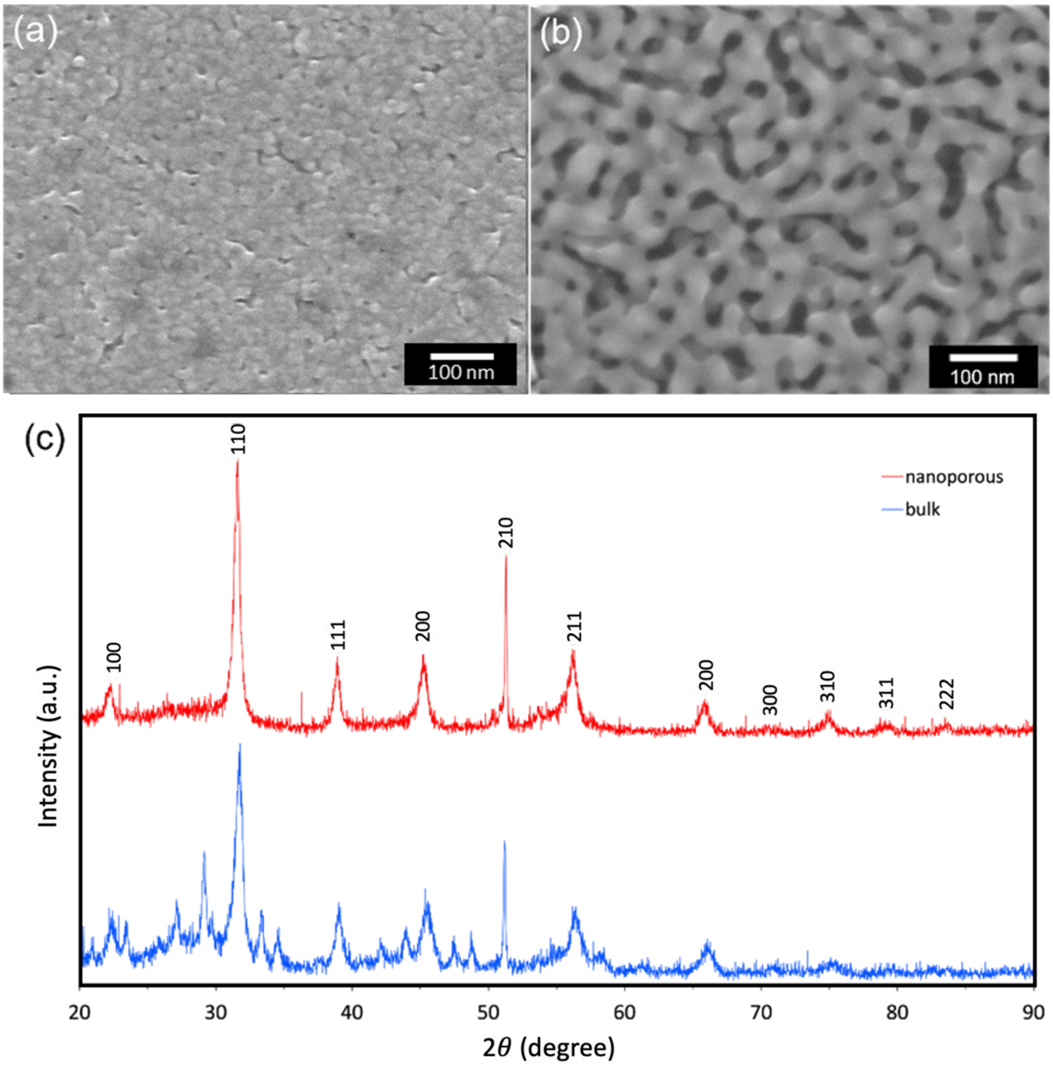

Figure S1. SEM image of the resulting films prepared (a) without PS-*b*-PEO (calcined at 700 ℃). (b) with PS-*b*-PEO (calcined at 650 ℃). (c) XRD patterns of the resulting films prepared without PS-*b*-PEO (calcined at 700 ℃) and with PS-*b*-PEO (calcined at 650 ℃). The incident angle is set to a minimum to avoid peaks from the 0.5 wt.% Nb-doped STO substrate.

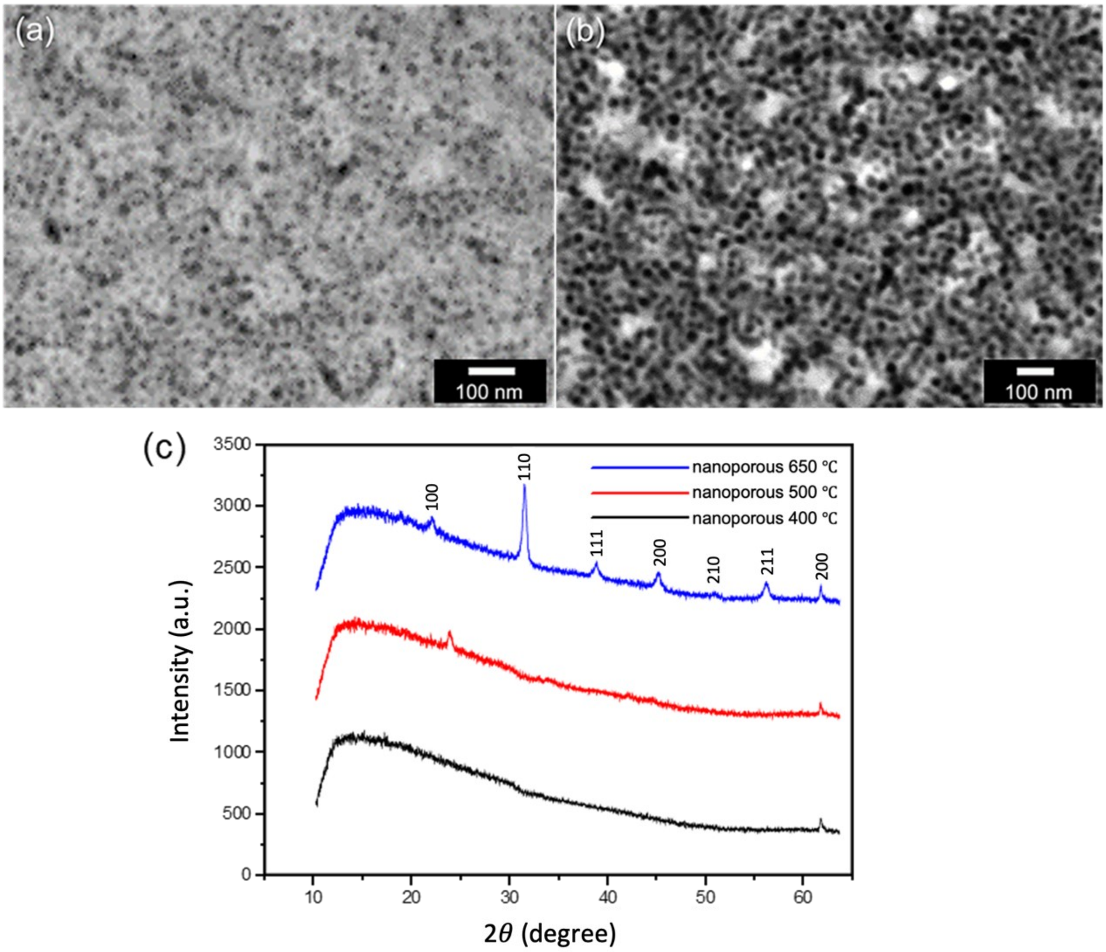
(a)
100 nm
(b)
100 nm
(c)
nanoporous 650 ℃
nanoporous 500 ℃
nanoporous 400 ℃
100
110
111
200
210
211
200
Intensity (a.u.)
2θ (degree)
0
500
1000
1500
2000
2500
3000
3500
10
20
30
40
50
60

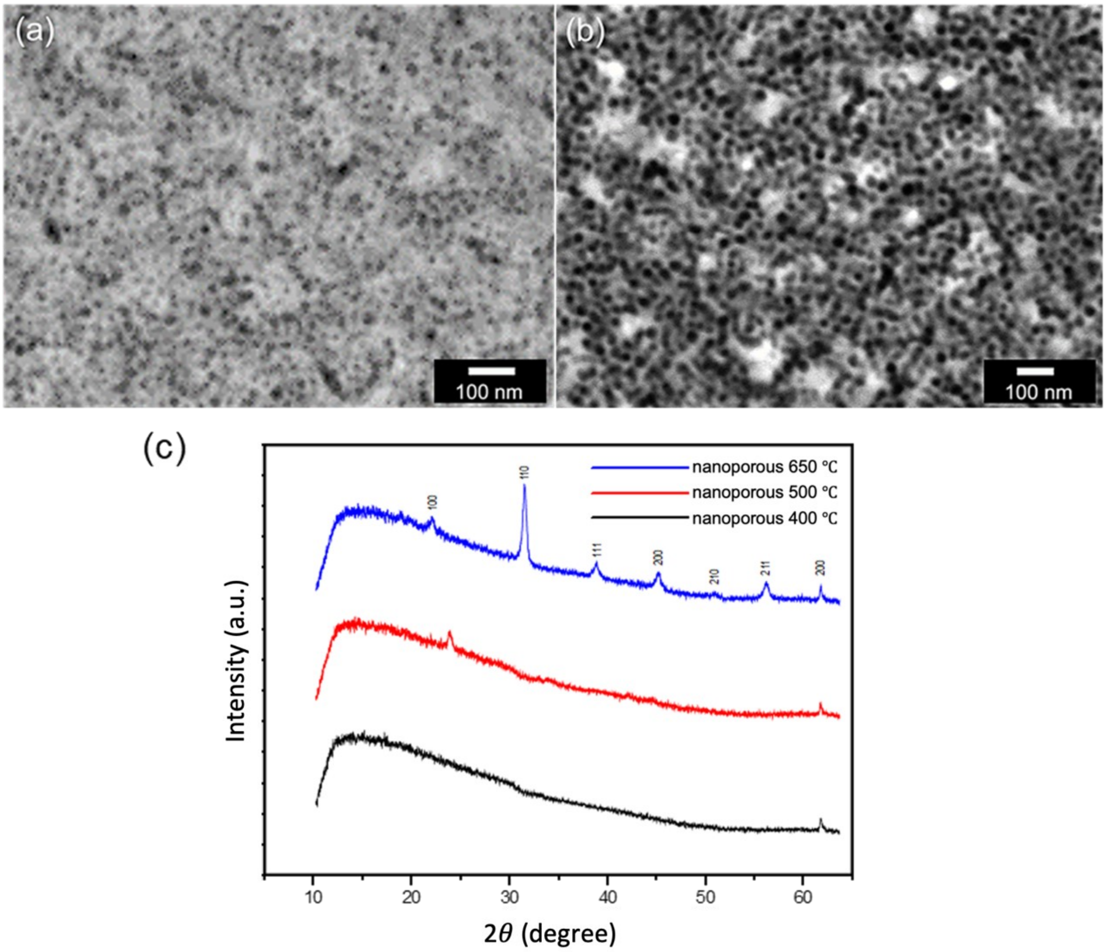

Figure S2. Top-view SEM images of the resulting nanoporous BCZT films calcined at (a) 400 ℃ and (b) 500 ℃. (c) Screening XRD at 400 ℃, 500 ℃ and 650 ℃

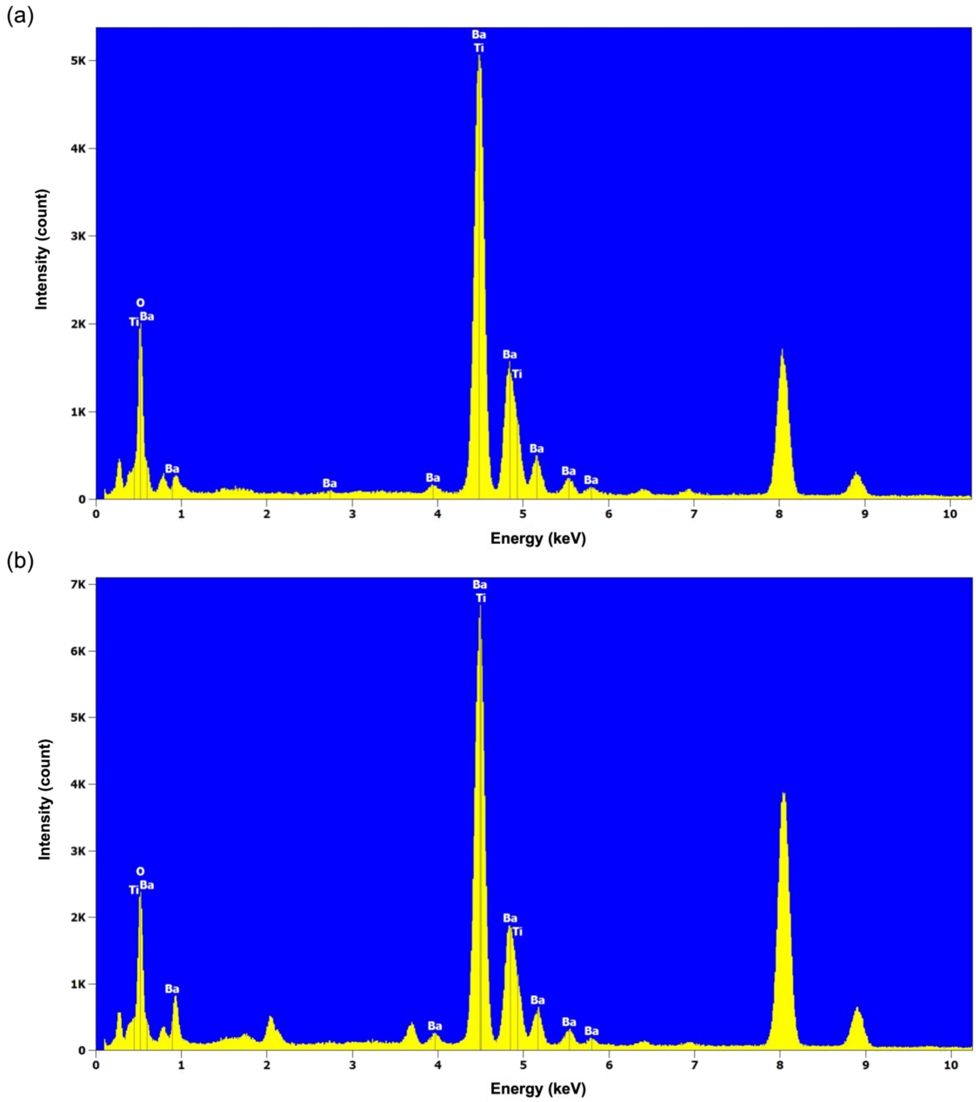

Figure S3. EDS spectra for nanoporous (a) BTO and (b) BCZT. The spectra for Ba, Ti and O are observed, although the spectra for Ti overlap with those for Ba in both samples.

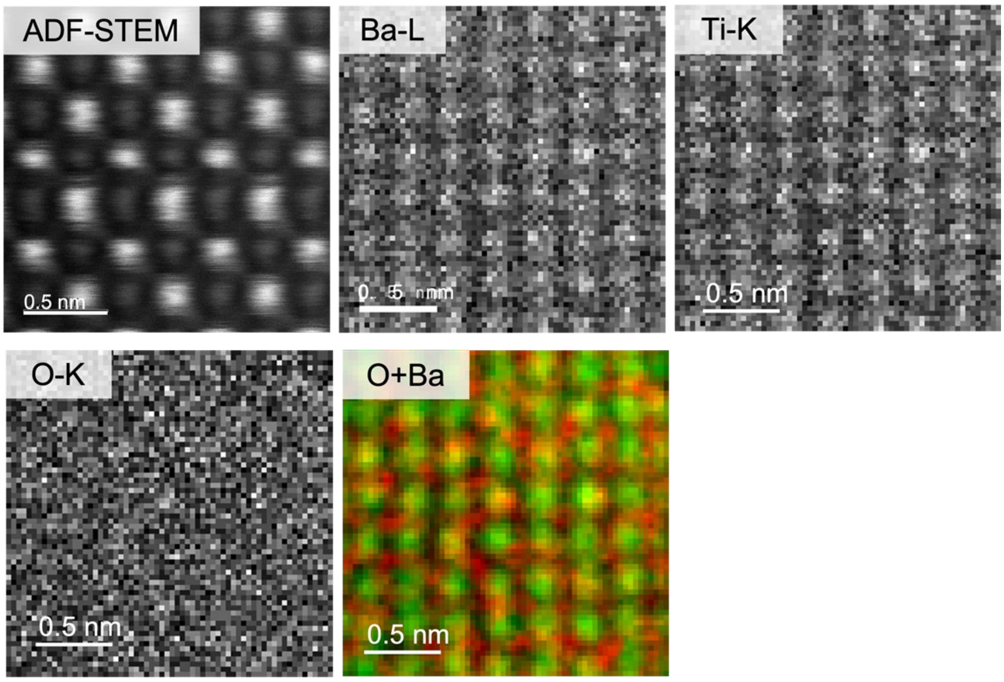

Figure S4. High-angle annular dark-field scanning transmission electron microscopy (HAADF-STEM) image, element distribution mapping, and RGB overlap mapping for the nanoporous BTO film.

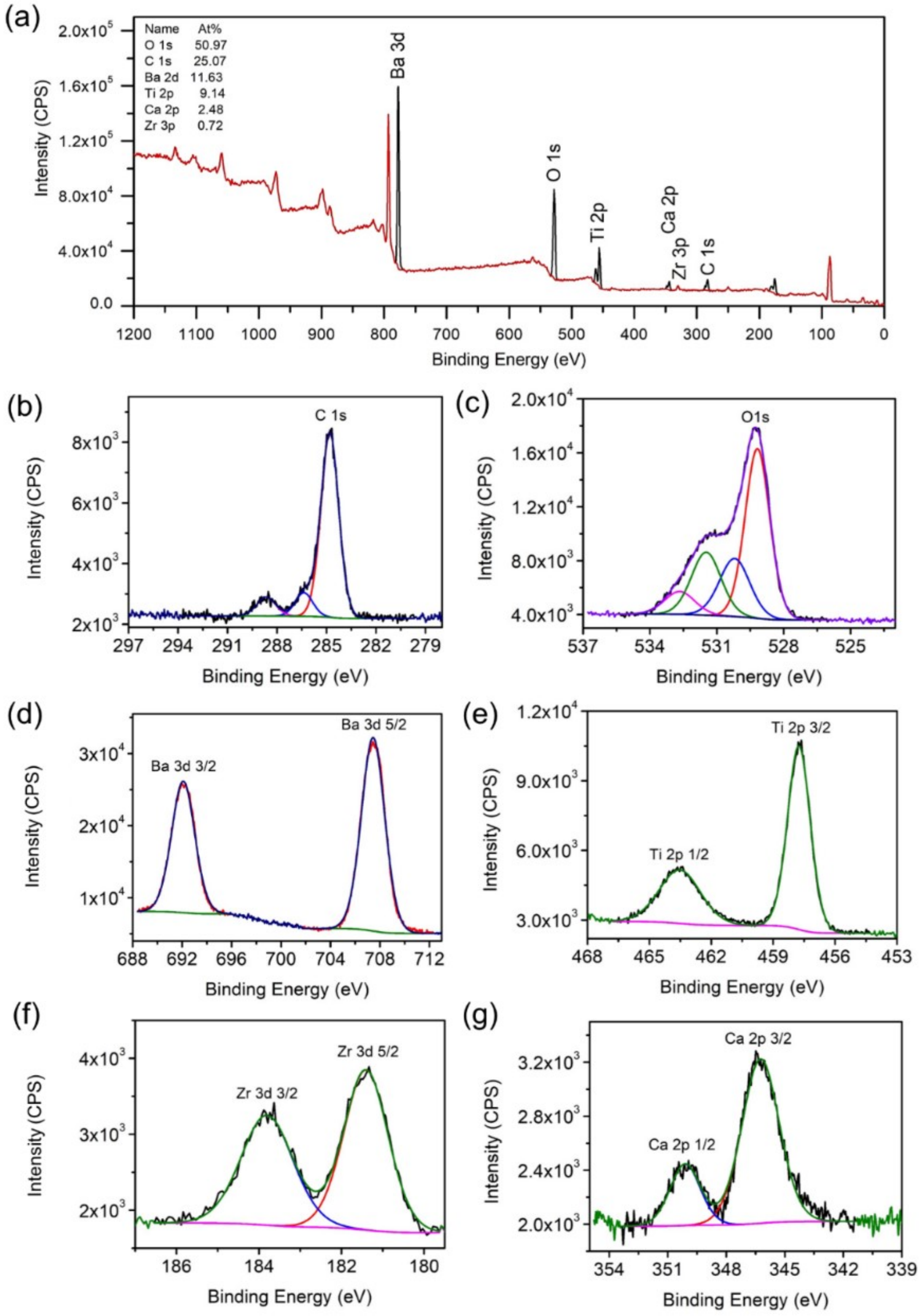

Figure S5. XPS spectra obtained from the surface of nanoporous BCZT calcined at 650 °C. (a) A wide-range spectrum. High-resolution spectra for (b) C, (c) O, (d) Ba, (e) Ti, (f) Zr and (g) Ca.

Comment for Figure S5: It is clear that the wide-range XPS spectra demonstrate the existence of predictable Ba, Ca, Zr, Ti, and O peaks (**Figure S5a**). Ba 3*d*, Ca 2*p*, Ti 2*p*, Zr 3*p,* and O 1*s* peaks with

compositions of 11.63, 2.48, 9.14, 0.72, and 50.97 mol% of the synthesized composite are observed, which confirms successful doping. The above calculation of the stoichiometric composition from the XPS result of nanoporous $Ba_{0.84}Ca_{0.16}(Ti_{0.93}Zr_{0.07})O_3$ clearly fits with the nominal composition of nanoporous $Ba_{0.85}Ca_{0.15}(Ti_{0.9}Zr_{0.1})O_3$ synthesized in this study. The XPS spectrum of C 1*s* is generally considered to indicate surface contamination of the synthesized film (**Figure S5b**). In the current analysis, the C 1*s* peak centered at 284.85 eV is the reference for calibrating the binding energies of the elements present in the BCZT composition. The asymmetrical high-resolution XPS peak of the O 1*s* orbital in each spectrum is deconvoluted into three different component peaks (**Figure S5c**). The peaks are centered at 529.15, 530.26, 531.25, and 532.58 eV for nanopores [4]. The peak centered at 529.15 eV corresponds to lattice oxygen ions (O^{2-}) in the perovskite structure, whereas the peak centered at 530.26 eV can be attributed to the O^{2-}/O^{-} ions caused by oxygen vacancies [5]. The peak centered at 531.25 eV is due to chemisorbed oxygen on the thin film surface, as reported earlier by Tu *et al*. [6] and Usman *et al*. [7] for ZnO nanorods and ZnO thin films, respectively. The satellite peak at 532.58 eV may be due to the overlap of orbitals. However, a slight shift in the binding energy for the O 1*s* spectral components in the BCZT thin films occurred due to different chemical environments and may also be due to charging during XPS experiments [8]. The high-resolution XPS spectra of the Ba 3*d* orbital exhibit two peaks at 709.12 eV and 693.6 eV, attributed to Ba $3d_{5/2}$ and Ba $3d_{3/2}$, respectively (**Figure S5d**), whereas the Ti 2*p* spectra exhibit two peaks at 457.75 and 463.5 eV, attributed to Ti^{4+} $2p_{3/2}$ and Ti^{4+} $2p_{1/2}$, respectively (**Figure S5e**) [9]. Moreover, Zr 3*d* exhibits two peaks at 181.25 and 183.75 eV, attributed to Zr $3d_{5/2}$ and Zr $3d_{3/2}$, respectively (**Figure 5f**). Ca 2*p* exhibits two peaks at 346.52 and 350.22 eV, attributed to Ca $2p_{3/2}$ and Ca $2p_{1/2}$, respectively (**Figure S5g**).

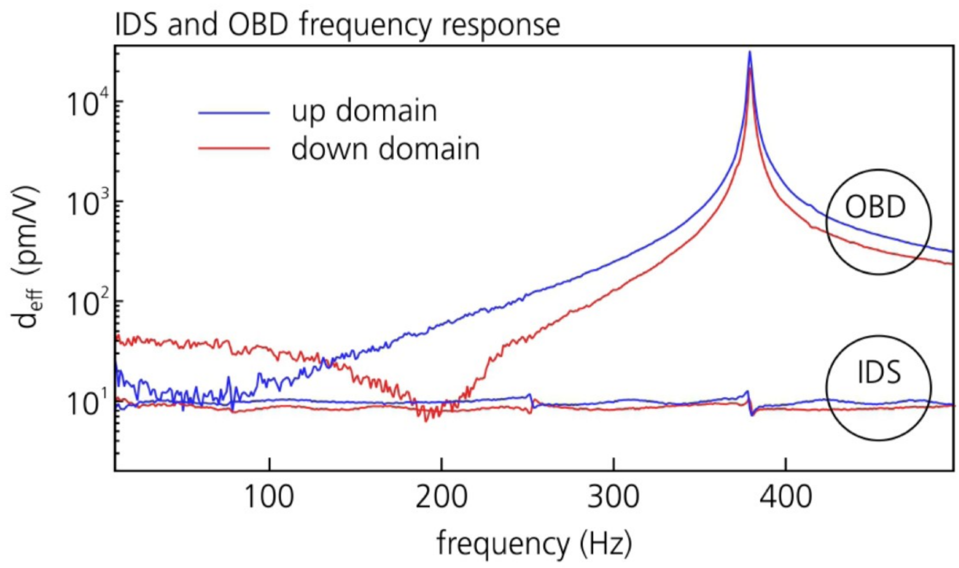

Figure S6. $G(\omega)$ Calibration reference. Calibration plot of proportionality factor $G(\omega)$ of optical beam deflection method (OBD) and interferometric displacement Sensor (IDS) for Cypher PFM (Source: *Oxford Instrument, USA*).[10]

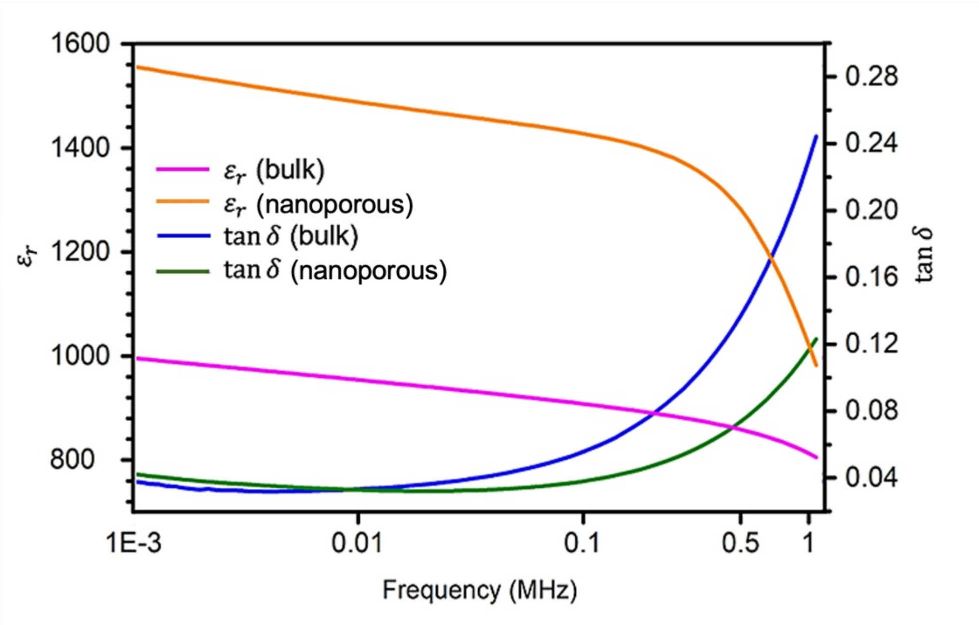

Figure S7. Dielectric constant and dielectric loss. Frequency-dependent ε_r and $\tan\delta$ for bulk and nanoporous BCZT value measured from 100 μm dia-disc area.

Comment for Figure S7: The ε_r and $\tan\delta$ values with frequency were measured for bulk and nanoporous BCZT films. ε_r for the nanoporous and bulk BCZT films shows a monotonous decrease with increasing frequency, although that for the nanoporous BCZT film shows an abrupt decrease above approximately 0.3 MHz. This is because, as frequency increases, the wavelength size approaches the size of the 'grains' the medium can no longer be represented as homogenous. Each transition of the wave between particles becomes a boundary condition, leading to dispersion and loss in the waves due to slight variations in the dielectric parameters of each particle. The ε_r values for the nanoporous film are 1.5 times larger than those for the bulk film up to 0.3 MHz. This higher ε_r value is attributed to the strain induced by nanopores [11,12]. On the other hand, the $\tan\delta$ values for both nanoporous and bulk films are almost the same at approximately 0.03 to 0.04 in the frequency range from 0.001 to 0.01 MHz, whereas they show an increase above 0.01 MHz. In addition, the $\tan\delta$ value for the nanoporous BCZT film shows a more gradual increase than that for the bulk BCZT film. It is approximately 0.12 at 1 MHz, which is approximately half that of the bulk film. Therefore, higher ε_r and lower $\tan\delta$ for the nanoporous film than for the bulk film are observed over this frequency range.

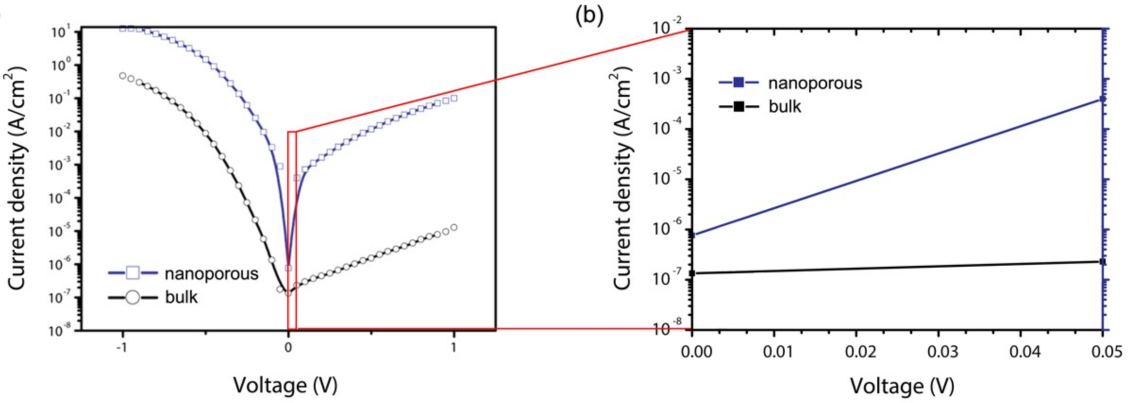

Figure S8. Leakage current. (a) *J-V* curves for bulk and nanoporous BCZT prepared on an Si/5 wt.% Nb-doped STO substrate at 650 ℃. (b) Magnified graph at a very low voltage marked in red in (a).

Comment for Figure S8: The leakage current density (*J*) is observed in both bulk and nanoporous BCZT films (. The peak current density values are 10^{-7} A cm^{-2} and 10^{-6} A cm^{-2} for the bulk and nanoporous BCZT films, respectively (**Figure S8a**). It is confirmed that the current density increases linearly with the applied voltage for both in the voltage range up to 0.05 V, although the current density for the nanoporous BCZT film is larger than that for the bulk film (**Figure S8b**). In our previous work, the current density of an STO/BTO hybrid single layer [12] was found to be 10^{-5} A cm^{-2} while maintaining a high dielectric constant. In a later work [13] on an STO/BTO multilayer stack, the current density was significantly reduced to 10^{-11} A cm^{-2}, but no significant ferroelectric properties were found. In the same context, a recent study by Mohamed *et al*. reported the synthesis of mesostructured HfO_2/Al_2O_3 composite thin films with a reduced leakage current of 10^{-9} A cm^{-2} at 1 V [14]. It is thought that the leakage current improvement may be caused by the effective stress at the interfaces between the mesoporous HfO_2 and Al_2O_3 domains, consequently affecting the stability of the mesostructured composite thin film. On the other hand, our nanoporous BCZT film reported here has an adequately low leakage current for use as a dielectric material. From the results of the dielectric properties and the leakage current measurements, a large dielectric constant is confirmed, and an adequately low leakage current is fit for practical use. Therefore, it is considered that nanoporous BCZT has more potential as a dielectric capacitor than bulk BCZT.

References

(1) Jesse, S.; Lee, H. N.; & Kalinin, S. V. Quantitative mapping of switching behavior in piezoresponse force microscopy. *Rev. Sci. Instrum*. **77**, 073702 (2006).

(2) Hÿtch, M. J., Putaux, J.L. & Pénisson, J. M. Measurement of the displacement field of dislocations to 0.03 Å by electron microscopy. *Nature*. **423**, 270–273 (2013).

(3) Labuda, A. & Proksch, R. Quantitative measurements of electromechanical response with a combined optical beam and interferometric atomic force microscope. *Appl. Phys. Lett.* **106**, 253103 (2015).

(4) Miot, C.; Husson, E.; Proust, C.; Erre, R.; Coutures, J. P. *J. Mater. Res.* **1997**, 12, 2388−2392.

(5) Reddy, S. R.; Prasad, V. V. B.; Bysakh, S.; Shanker, V.; Hebalkar, N.; Roy, S. K. Superior energy storage performance and fatigue resistance in ferroelectric BCZT thin films grown in an oxygen-rich atmosphere. *J. Mater. Chem. C.* **2019**, 7, 7073−7082.

(6) Tu, Y.; Chen, S.; Li, X.; Gorbaciova, J.; Gillin, W. P.; Krause, S.; Briscoe, J. Control of oxygen vacancies in ZnO nanorods by annealing and their influence on ZnO/PEDOT:PSS diode behaviour. *J. Mat. Chem. C.* **2018**, 6, 1815−1821.

(7) Ilyas, U.; Rawat, R. S.; Tan, T. L.; Lee, P.; Chen, R.; Sun, H. D.; Fengji, L.; Zhang, S. Oxygen rich *p*-type ZnO thin films using wet chemical route with enhanced carrier concentration by temperature-dependent tuning of acceptor defects. *J. Appl. Phys.* **2011**, 110, 093522.

(8) Craciun, V.; Singh, R. K., Characteristics of the surface layer of barium strontium titanate thin films deposited by laser ablation. *Appl. Phys. Lett.* **2000**, 76, 1932−1934.

(9) Wei, K.; Wang, B.; Hu, J.; Chen, F.; Hao, Q.; He, G.; Wang, Y.; Li, W.; Liu, J.; He, Q. Photocatalytic properties of a new Z-scheme system $BaTiO_3/In_2S_3$ with a core–shell structure. *RSC Adv.* **2019**, 9, 11377−11384.

(10) Oxford Instruments, Interferometric Displacement Sensor Option for the Cypher AFM. *Datasheet for Interferometric Displacement Sensor*, **2018**, 1-4.

(11) Suzuki, N.; Zakaria, M. B.; Torad, N. L.; Wu, K. C.-W.; Nemoto, Y.; Imura, M.; Osada, M.; Yamauchi, Y. Synthesis of Highly Strained Mesostructured $SrTiO_3/BaTiO_3$ Composite Films with Robust Ferroelectricity. *Chem. Eur. J.* **2013**, 19, 4446−4450.

(12) Suzuki, N.; Jiang, X.; Salunkhe, R. R.; Osada, M.; Yamauchi, Y. Chemical Preparation of Ferroelectric Mesoporous Barium Titanate Thin Films: Drastic Enhancement of Curie Temperature Induced by Mesopore-Derived Strain. *Chem. Eur. J.* **2014**, 20, 11283−11286.

(13) Zakaria, M. B.; Nagata, T.; Matsuda, A.; Yasuhara, Y.; Ogura, A.; Hossain, M. S. A.; Billah, M.; Yamauchi, Y.; Chikyow, T. *ACS Appl. Nano Mater.* **2018**, 1, 915–921.

(14) Zakaria, M. B.; Nagata, T.; Chikyow, T. Mesostructured HfO_2/Al_2O_3 Composite Thin Films with Reduced Leakage Current for Ion-Conducting Devices. *ACS Omega.* **2019**, 4, 14680–14687.
